# Prevalence of cardiometabolic risk factors among professional male long–distance bus drivers in Lagos, south–west Nigeria: a cross–sectional study 

**DOI:** 10.5830/CVJA-2018-006

**Published:** 2018

**Authors:** E Amadi Casmir, C Mbakwem Amam, B Ozoh Obianuju, P Grove Tim, A Wood David, A Kushimo Oyewole, Akinkunmi Michael

**Affiliations:** Department of Medicine, College of Medicine, University of Lagos, Nigeria; Department of Medicine, College of Medicine, University of Lagos, Nigeria; Department of Medicine, College of Medicine, University of Lagos, Nigeria; National Heart and Lung Institute, Imperial College, London; National Heart and Lung Institute, Imperial College, London; Department of Medicine, Lagos University Teaching Hospital, Nigeria; Department of Medicine, Lagos University Teaching Hospital, Nigeria

**Keywords:** cardiovascular disease, risk factors, long–distance drivers

## Abstract

**Background:**

Professional drivers are known to be at high risk of cardiovascular disease (CVD). This study was carried out to highlight these risk factors and their predictors among male long–distance professional bus drivers in Lagos, southwest Nigeria, with a view to improving health awareness in this group.

**Methods:**

Socio–demographic data, anthropometric indices, blood pressure, fasting plasma blood glucose levels and lipid and physical activity profiles of 293 drivers were measured.

**Results:**

Mean age of the study population was 48 ± 9.7 years; 71.0 and 19.5% of the drivers used alcohol and were smokers, respectively; and 50.9% were physically inactive. The prevalence of overweight and obesity was 41.7 and 21.1%, respectively, while 39.7 and 13.9% were hypertensive and diabetic, respectively. Ninety (31.3%) subjects had impaired fasting glucose levels while 56.3% had dyslipidaemia. Predictors of hypertension were age and body mass index (BMI). BMI only was a predictor of abnormal glucose profile.

**Conclusion:**

Professional male long–distance bus drivers in this study showed a high prevalence of a cluster of risk factors for CVD.

Atherosclerotic cardiovascular disease (CVD), typified by coronary heart disease (CHD) and stroke, is a pre–eminent cause of preventable and premature mortality globally, accounting for about 30% of global deaths.^1^ This is expected to increase by almost 50% by 2030.^2^ It is also a major cause of mass disability and a somatic cause of loss of productivity globally, with over 150 million disability adjusted life years (DALYS).^3^ About 80% of this burden from CVD is borne by low– and middle–income countries (LMIC).^1^

Globally, CVD prevalence is on the increase, remarkably so in the LMIC. This is largely due to increased urbanisation and its corollary of better socio–economic opportunities and Westernisation of lifestyles, such as sedentary living, unhealthy dietary choices, tobacco use, psycho–social stress and harmful use of alcohol.^4^ These behavioural risk factors predispose to intermediary or metabolic risk factors, such as hypertension, abnormalities in blood glucose levels, dyslipidaemia, overweight and obesity.^5^,^6^

One of the socio–economic consequences of urbanisation is mass transit of people, goods and services across regions and long distances via land, air and waterways. The consequence of this is the creation of effective road transport systems in urban areas, with an increase in the number of people engaged in professional driving.

Professional drivers as an occupational group are at increased risk of CVD. Morris et al., in their seminal research in 1953, documented that London bus drivers were at increased risk for CHD compared to the more active bus conductors.^7^ Several other occupational epidemiological studies have provided evidence that professional drivers (short– and long–distance drivers) suffer more and die from CVD.^8^–^11^ This excess of CVD morbidity and mortality risk among this group is attributable to a high prevalence of CVD risk factors, such as obesity, hypertension, sedentary living, diabetes, smoking and unhealthy diets found in them.^12^–^14^

Beyond these conventional risk factors for CVD, various driving–related activities, such as traffic congestion, ergonomic factors, long–distance driving, shift work, and anxiety and tension from the job of driving have also been implicated. These are known to cause various neuroendocrine and neurocardiological responses, such as increased secretion of cortisol and catecholamines, and decreased heart rate variability, which may also be possible mediators of CVD.^15^,^16^ They can also be considered a vulnerable group with social gradients of inequalities; they usually belong to the lower socio–economic class, are not well educated/informed and are not usually covered by public health policies. They also work under immense anxiety and stress. These further heighten their risk for CVD.

Lagos is the second most populous city in Nigeria, the second fastest growing city in Africa and the seventh in the world.^17^ It is the economic hub of the country with well–developed intra–city, inter–city and trans–African highway routes for easy mass transit of people, goods and services across geographical barriers,^18^ making road transportation and the transportation business important features of its economy. Therefore many companies engage in long–distance transportation, with professional drivers employed to provide this service.

In Nigeria there are few studies on the CVD risk profile of this important but vulnerable group. These studies show that long–distance drivers have a significant burden of hypertension and overweight/obesity, comparable to or even higher than in the general population.^19^–^21^ Hypertension is a common and important CVD risk factor. Its prevalence among long–distance bus drivers in Nigeria is 22.5%,^19^ which was also the pooled prevalence of hypertension in the general population in 2012.^22^ However, none of these studies screened the drivers for diabetes/ abnormal glucose profiles or dyslipidaemia.

Considering the potential risk associated with professional driving, the importance of bus drivers to the country’s socio–economic development and the paucity of data on the cardiovascular risk profile of long–distance bus drivers, it became necessary to investigate the prevalence of cardiometabolic and lifestyle–related risk factors for CVD and their predictors in this segment of the Nigerian working population in Lagos, southwest Nigeria. The findings from this study will also help create awareness of their risk burden and possibly help shape policies to address this risk.

## Methods

This was a cross–sectional study involving male long–distance bus drivers in major motor parks in Lagos. The parks were selected based on their size and the routes they serve. Long–distance driving was defined as a distance of 160–km radius from the terminal of departure.^23^

The calculated sample size was 268 based on the prevalence of hypertension in the general population.^22^ To allow for 15% attrition rate, the sample size was increased to 308. However, 15 of the drivers did not have complete data and were not included in the data analysis, giving a response rate of 95%. Therefore 293 was the final sample size used in the data analysis. Ethical approval for the study was obtained from the Health Research Ethics Committee of the Lagos University Teaching Hospital.

We used a stratified cluster–sampling method to recruit longdistance drivers registered with the Transport Workers’ Union from selected motor parks in Lagos between March and July 2015. The motor parks were then stratified based on whether or not they organised mandatory annual health and safety training for their drivers (AHS motor parks). Only two motor parks employing 400 drivers met this criterion. The drivers in the AHS motor parks only operate from their company terminals. We selected one of these for inclusion in the study because its annual health and safety programme coincided with the study period. All 168 drivers agreed to participate but three (1.8%) later declined.

**Fig. 1:**
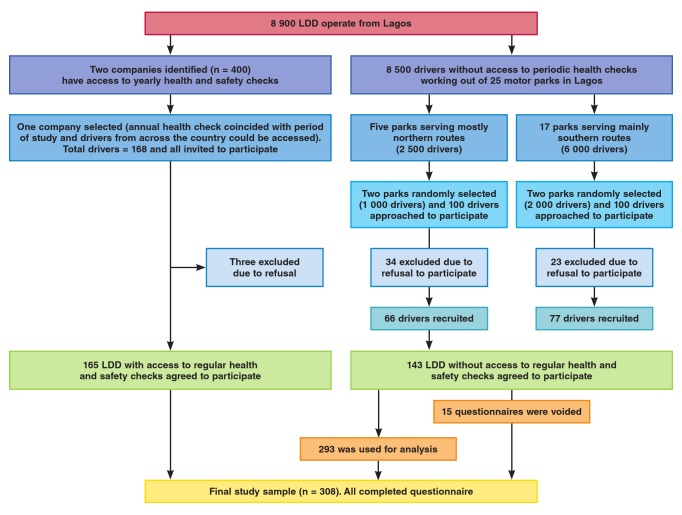
Consort diagram describing how participants were recruited into the study. LDD: long-distance commercial drivers.

The second category of (non–AHS) motor parks comprised independent drivers and drivers working for small transport companies that operate from general and less regulated motor parks in Lagos and who do not routinely receive formal health and safety checks. We divided these motor parks into two; those serving the northern and southern parts of the country, respectively. We then randomly selected two motor parks from each of these strata for inclusion in the study, thereby selecting four in total. Finally, we used a convenience sample of 50 drivers from each of these four parks and recruited 143 of them (71.5% response rate). Those who declined did so due to time constraints and undisclosed personal reasons. Fig. 1 shows the consort diagram on how the participants were recruited.

On a mutually agreed day, the consenting drivers were approached in groups and were given a talk on the importance of healthy living and they were also briefed on the usefulness of the study. They were told to observe an overnight fast on the day of the medical screening. We used a structured questionnaire administered by trained interviewers to obtain their sociodemographic data and relevant medical history. Those who couldn’t read or write were assisted to complete the questionnaire by interviewers who could speak their native languages.

Thereafter their body weights were measured in kilograms with an Omron HN289 (Osaka, Japan) digital weighing scale, placed on a firm, flat ground, with participants wearing light clothing and with no footwear or cap. Measurements were taken to the nearest 0.5 kg, after ensuring that the scale was always at the zero mark.

Their heights were measured in centimetres with a Seca model 216 (GmbH, Hamburg, Germany) stadiometer with the participant standing erect, back against the height metre rule and occiput and heels making contact with the height metre rule. BMI was calculated as weight in kilograms divided by height squared in metres.^24^ BMI was categorised as underweight < 18.0 kg/m^2^; normal weight 18.0–24.9 kg/m^2^; overweight 25.0–29.9 kg/ m^2^; class I obesity 30.0–34.9 kg/m^2^; class II obesity 35.0–39.9 kg/ m^2^ and class III obesity > 40.0 kg/m^2^.

Participants’ waist circumferences were measured with an inextensible, inelastic 1–cm–wide tape snug around the body at the level of the midpoint between the lower margin of the last palpable rib and the top of the anterior iliac crest. Measurements were taken at the end of normal respiration and ≥ 102 cm was regarded as abdominal obesity.^25^ Their neck circumferences were also measured with an inextensible, inelastic 1–cm–wide tape at the level of the cricoid cartilage. A neck circumference ≥ 40 cm defined obesity.^26^

The blood pressure (BP) of the participants was measured by the research assistants after five minutes of rest, with the participant seated comfortably, feet on the floor, arm at the level of the heart and free of any constricting clothing. Appropriate–sized cuffs and bladder connected to an Omron HEM7233 (Osaka, Japan) digital sphygmomanometer were used in measuring the BP, which was taken initially on both arms, and the arm with the higher value was used in subsequent measurements. Three BP readings were taken at two– to three–minute intervals. The average of three readings was taken for analysis. Hypertension was defined as BP ≥ 140/90 mmHg, self–volunteered history of hypertension and/or use of anti–hypertensives.

Venepuncture was done on each participant while observing aseptic techniques. Five millilitres of venous blood was put in fluoride oxalate and lithium heparin vacutainer specimen bottles for fasting plasma glucose and fasting lipid profiles, respectively, and sent to the laboratory for processing and analysis with a Beckman (Pasadena, CA, USA) automated clinical chemistry autoanalyser using standard reagents/kits from Randox Laboratories.^27^ Participants with a fasting plasma glucose value of ≥ 126 mg/dl (6.99 mmol/l), self–volunteered history of diabetes and or use of insulin/oral hypoglycaemic agents were regarded as diabetic, while a fasting plasma glucose level between 100 and 125 mg/dl (5.55–6.94 mmol/l) was regarded as impaired fasting glucose.^28^ For the purpose of this study, abnormal glucose profile was defined as a combination of impaired fasting glucose and frank diabetes.

Abnormal lipid profile was determined from the ATP III guidelines of 2001; total cholesterol (TC) ≥ 240 mg/dl (6.22 mmol/l), high–density lipoprotein cholesterol (HDL–C) ≤ 40 mg/ dl (1.04 mmol/l), and low–density lipoprotein cholesterol (LDLC) > 160 mg/dl (4.14 mmol/l) and triglycerides > 150 mg/dl (1.70 mmol/l).29 Atherogenic dyslipidaemia was defined by the Castelli index as TC/HDL–C > 3.4.^30^

The physical activity level of participants was assessed with the World Health Organisation (WHO) Global Physical Activity Questionnaire–2 (GPAQ–2), which assesses physical activity in four domains of work, travel, recreational and resting.31 The product of the exercise intensity in metabolic equivalents (METs), duration of activity in hours and the number of times per week, expressed as METs/hour was regarded as exercise volume. A MET/hour value less than 600 per week was taken as physical inactivity.^31^

## Statistical analysis

Data entry and analysis were done with the Statistical Package for the Social Sciences 17.0 version (SPSS, Inc, Chicago, IL, USA). Continous data are presented as mean and standard deviation. Categorical variables are expressed as proportions. Pearson’s correlation was used to determine how some independent numerical variables (age, BMI, number of years of professional driving and number of driving hours/week) correlated with the major outcome variables (systolic and diastolic BP, and abnormal glucose profile). Furthermore, the independent variables were dichotomised to look for an association between them and the outcome variables, hypertension and abnormal glucose profile using the chi–squared test. Level of statistical significance was set at p < 0.05 and confidence interval at 95%.

Multivariate analysis was done using a forward stepwise binary logistic regression in order to assess for independent predictors of hypertension and abnormal glucose. We included predictor variables with associations at a significance level of p ≤ 0.2 on univariate analysis in order to accommodate for important risk factors.

## Results

A total of 308 drivers were recruited for the study. Fifteen were excluded due to incomplete data. Therefore 293 were used for data analysis, giving a response rate of 95.1%.

The age range of the study population was between 25 and 76 years with a mean of 44.8 ± 9.7 years. Two hundred and eightysix (97.6%) of the subjects were aged between 25 and 65 years. The rest of their socio–demographic characteristics is shown in Table 1.

Fifty–seven of the drivers (19.5%; 95% CI: 14.9–24.0%) were active smokers while 217 (74.1%) and 19 (6.5%) were non–smokers and ex–smokers, respectively. The prevalence of alcohol intake was 71.1% (95% CI: 65.7–76.2%). The majority consumed various types of alcoholic beverages: beer, spirits and alcohol–based herbal medications. The intake of alcohol was about four bottles of beer per week (Table 1).

**Table 1:** Socio-demographic characteristics of the subjects

*Parameters*	*Mean ± SD*	*n (%)*
Age (years)	44.8 ± 9.7	
25–44		147 (50.2)
45–64		139 (47.4)
> 65		7 (2.4)
Educational level		
Primary		77 (26.3)
Secondary		177 (60.4)
Tertiary		37 (12.6)
Marital status		
Married		265 (90.4)
Single		22 (7.5)
Widowed		3 (1.0)
Divorced		3 (1.0)
Number of years as a professional driver	20.0 ± 10.4	
Number of hours driven per week	41.9 ± 28.7	
Smoking pattern		
Active smokers		57 (19.5)
Non-smokers		217 (74.1)
Ex-smokers		19 (6.5)
Alcohol use		
User		208 (71.1)
Teetotaler		85 (29.0)

**Fig. 2:**
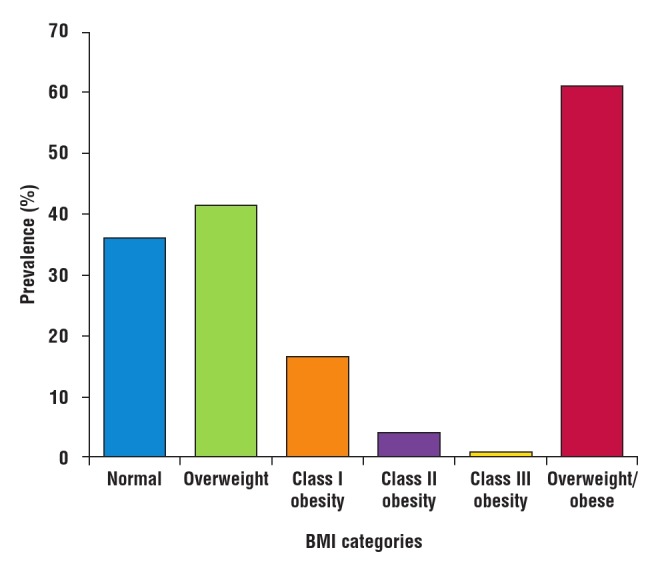
Prevalence of the various categories of BMI.

The mean BMI of the subjects was 27.2 ± 9.6 kg/m^2^, with 121 (41.7%) and 61 (21.1%) being in the overweight and obese categories, respectively. The prevalence of overweight and obesity were 41.7% (95% CI: 36.0–47.4%) and 21.1% (95% CI: 16.3–25.6%), respectively, giving a combined prevalence of 62.8% (95% CI: 57.2–68.3%) (Table 2). Fig. 2 shows the frequency of the various classes of obesity.

**Table 2:** Measures of obesity, BP and glucose profile of the subjects

*Parameter*	*Mean ± SD*	*n (%)*
BMI (kg/m^2^)	27.2 ± 9.6	
Waist circumference (cm)	96.4 ± 0.9	
Proportion < 102 cm		168 (66.4)
Proportion ≥ 102 cm		125 (43.3)
Neck circumference (cm)	39.2 ± 2.8	
Proportion < 40 cm		171 (59.6)
Proportion ≥ 40 cm		131 (41.6)
Blood pressure		
SBP (mmHg)	136.3 ± 20.9	
DBP (mmHg)	83.2 ± 13.6	
Total number of hypertensives		116 (39.7)
Newly diagnosed		88 (75.9)
Previously known hypertensives		29 (9.6)
Blood glucose		
Fasting blood glucose (mg/dl)	108.2 ± 39.7	
Normoglycaemia		158 (54.9)
Impaired fasting glucose		90 (31.3)
Total number of diabetics		40 (13.9)
Newly diagnosed diabetics		33 (82.5)
Previously known diabetics		7 (17.5)

SBP: systolic blood pressure; DBP: diastolic blood pressure.

The mean waist circumference (WC) of the study population was 94.9 ± 11.9 cm, while the prevalence of abdominal obesity,WC ≥ 102 cm, was 24.1% (95% CI: 19.2–29.0%). The mean neck circumference of the study population was 39.2 ± 2.8 cm, with 28.8% having a neck circumference ≥ 40 cm (Table 2).

The mean systolic blood pressure (SPB) and diastolic blood pressure (DBP) of the subjects were 136.3 ± 20.9 and 83.2 ± 13.6 mmHg, respectively. One hundred and sixteen cases of hypertension were identified, giving a prevalence rate of 39.7% (95% CI: 34.0–45.25%). Eighty–eight (75.9%) were detected for the first time during the study. Twenty–eight (24.1%) were previously known hypertensives, with six (21.4%) having good BP control (Table 2).

The mean fasting blood glucose level (FBG) of the study population was 108.2 ± 39.7 mg/dl (6.01 ± 2.2 mmol/l). Forty of the subjects (13.9%; 95% CI: 9.7–17.6%) had diabetes and seven (2.4%) were previously known diabetics. Ninety (31.3%) had impaired fasting glucose levels. Prevalence of abnormal glucose profiles (diabetes + impaired FBG) was 45.2% (95% CI: 39.3–50.7%) (Table 2).

The mean TC of the study population was 218.4 ± 33.2 mg/ dl (5.66 ± 0.86 mmol/l). The overall lipid profile is presented in Table 3. One hundred and twenty–eight (43.7%) of the subjects had normal lipid profiles while 165 (56.3%) had one form of dyslipidaemia or another. The prevalence of dyslipidaemia in the study was 56.3% (95% CI: 50.6–62.0%), while the prevalence of atherogenic dyslipidaemia, i.e. elevated TC/HDL–C was 33.1% (95% CI: 27.7–38.5%) (Table 3).

**Table 3:** Pattern of lipid profiles of the subjects

	*Mean ± SD*	
*Parameter*	*mg/dl*	*mmol/l*	*n (%)*
TC	218.4 ± 33.2	5.66 ± 0.86	
LDL-C	136.4 ± 33.6	3.53 ± 0.87	
HDL-C	57.7 ± 15.3	1.49 ± 0.40	
TG	122.7 ± 64.1	1.39 ± 0.72	
Non-HDL-C	161.0 ± 31.5		
TC/HDL-C	3.8 ± 1.9		
TG/HDL	3.7 ± 2.6		
Abnormal profiles
Elevated TC			81 (27.8)
Elevated LDL-C			72 (24.6)
Low HDL-C			19 (6.5)
Elevated TC/HDL-C			96 (33.1)
Elevated TG/HDL-C			38 (13.0)

TC: total cholesterol; LDL-C: low-density lipoprotein cholesterol; HDL-C: high-density lipoprotein cholesterol; TG: triglycerides.

**Fig. 3:**
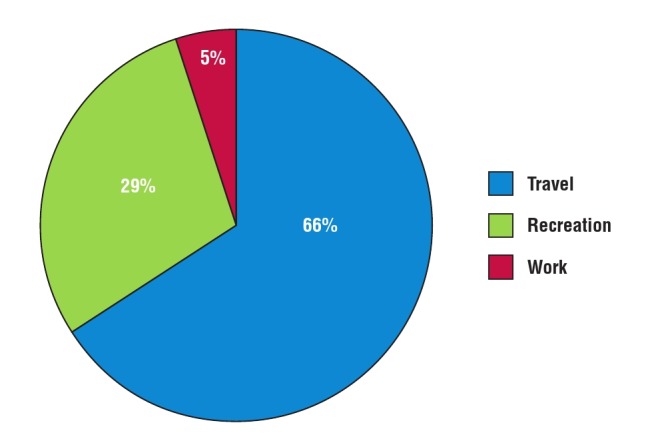
Contributions of the GPAQ2 domains to total physical activity of the subjects.

The mean METs/hour of the subjects was 638.8 ± 565.5, with 66% of them spending most of their time in the travel domain (Fig. 3). The prevalence of physical inactivity in the study population, defined as total METs/hour in all four domains < 600 per week was 50.9% (95% CI: 53.1–64.3%). Two hundred and thirty–four (80.4%) of the subjects were inactive, 56 (19.2%) were low active, while one (0.3%) was medium active. None was highly active.

**Fig. 4:**
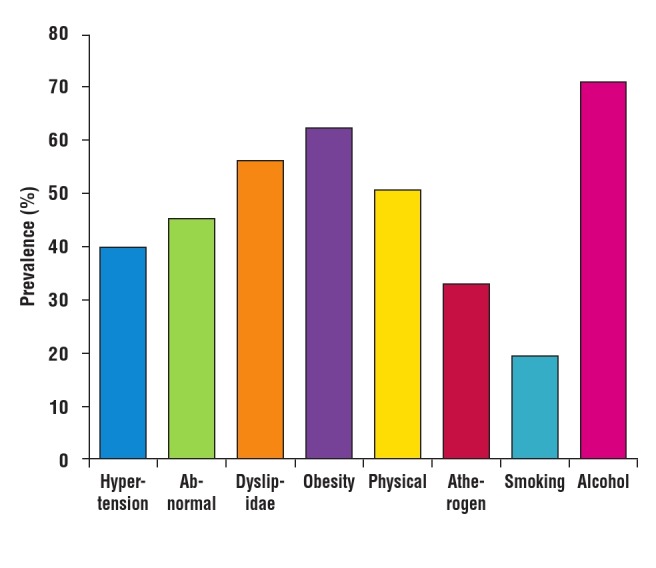
Prevalence of single risk factors among the subjects

**Fig. 5:**
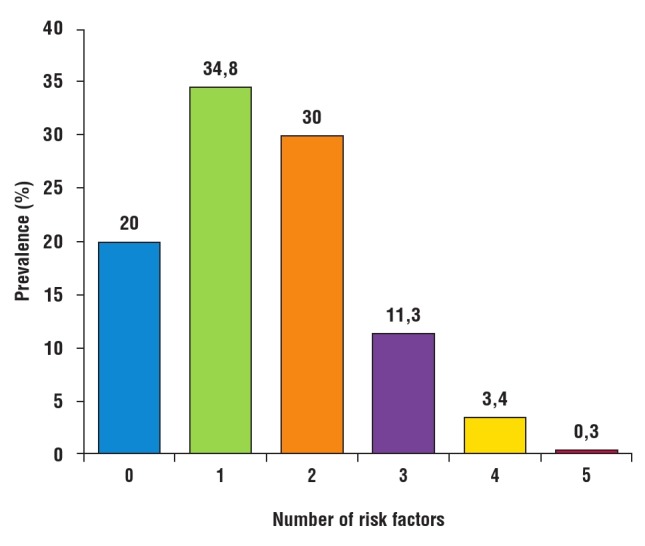
Prevalence of multiple risk factors among the subjects.

**Fig. 6:**
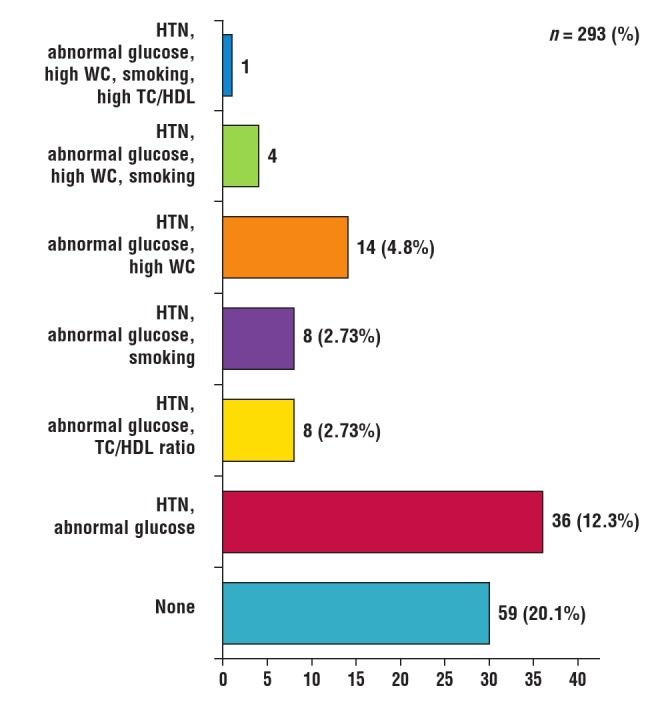
Prevalence of different combinations of risk factors in the subjects. HTN: hypertension; WC: waist circumference; TC: total cholesterol; HDL: high–density lipoprotein cholesterol.

One hundred and thirty–two (45.1%) subjects had co–occurrence of two or more risk factors. The most prevalent combination was the duo of hypertension and abnormal glucose profile. Fig. 4 shows the common single risk factors, while common risk factor combinations are shown in Fig. 5. Alcohol use and physical inactivity were the commonest behavioural risk factors, while overweight/obesity, hypertension and dyslipidaemia were the three most common metabolic risk factors in the subjects (Fig. 6).

Pearson’s correlation was used to determine how some independent numerical variables (age, BMI, number of years of professional driving and number of driving hours/week) correlated with the major outcome variables (SBP, DBP and fasting blood glucose level). Age correlated significantly with SBP (r = 0.362, p < 0.001) and DBP (r = 0.335, p < 0.001). BMI also correlated significantly with SBP (r = 0.288, p < 0.001) and DBP (r = 0.208, p < 0.001). BMI alone correlated significantly with fasting glucose (r = 0.136, p = 0.021).

Furthermore, the independent variables were dichotomised to look for an association between them and outcome variables of hypertension and abnormal glucose profile. In this model only age, BMI, number of years of professional driving and waist circumference had significant associations with hypertension, while none of these except BMI had a significant association with abnormal glucose levels (Table 4).

**Table 4:** Association between independent variables and hypertension and abnormal glucose levels

	*Hypertension*		*Abnormal glucose levels*	
*Parameter*	*% (95% CI)*	*p-value*	*% (95% CI)*	*p-value*
Driving hours/week		0.250		0.076
≥ 36	42.9 (35.0–50.9)		35.6 (27.9–43.2)	
< 36	36.3 (28.5–44.2)		25.9 (18.6–33.2)	
Years of professional driving		< 0.001		0.320
≥ 20	56.2 (43.1–64.4)		33.1 (25.4–40.8)	
< 20	23.1 (16.2–30.0)		27.7 (20.3–35.0)	
Physical activity		0.279		0.205
< 600 METs/week	42.6 (34.6–50.5)		27.6 (20.3-34.9)	
≥ 600 METs/week	36.3 (28.5–44.2)		34.5 (26.7-42.3)	
BMI		< 0.001		0.002
Overweight/obese	48.4 (41.1–55.6)		37.8 (30.7–44.9)	
Normal	25.9 (17.7–34.2)		19.8 (12.2–27.4	
Alcohol use		0.840		0.807
Yes	40.1 (33.4–46.8)		31.2 (24.9–37.6)	
No	38.8 (28.5–49.2)		29.8 (20.0–39.5)	
Smoking		0.477		0.808
Yes	43.9 (31.0–56.7)		32.1 (19.9-44.4)	
No	38.7 (28.5-49.2)		30.5 (24.6-36.4)	
WC (cm)		< 0.001		0.076
> 102	61.4 (50.0–72.8)		39.7 (28.1–51.3)	
≤ 102	33.0 (26.8–39.2)		28.3 (22.3–34.3)	
Age		< 0.001		0.499
≥ 45	54.5 (46.4–62.6)		32.6 (25.0–40.3)	
< 45	25.2 (18.2–32.2)		29.0 (21.6–36.3)	

BMI: body mass index; WC: waist circumference; METs: metabolic equivalents

Multivariate analysis was done using a forward stepwise binary logistic regression in order to assess for independent predictors of hypertension and abnormal blood glucose levels. We included predictor variables with associations at a significance level of p ≤ 0.2 on univariate analysis in order to accommodate for important risk factors. The final logistic regression model (Table 5) showed that as age and BMI increased, the chances of becoming hypertensive increased 1.09 and 2.99 times (OR 1.09; 95% CI: 1.06–1.1, p < 0.0001; OR 2.99; 95% CI: 1.69–5.31, p < 0.0001), respectively.

For abnormal glucose level with increasing BMI, the chances of having abnormal glucose level increased 2.5 times (OR 2.39; 5% CI: 1.33–4.30; p = 0.004). Other variables such as physical activity, number of driving hours, waist circumference and professional driving years were not independently associated with our outcome parameters and were excluded from the final regression model.

**Table 5:** Logistic regression on predictors of hypertension and abnormal glucose levels

	*Hypertensiona*		*Abnormal glucose levelsb*	
*Variables*	*OR (95% CI)*	*p-value*	*OR (95% CI)*	*p-value*
Age	1.090 (1.058–1.23)	< 0.0001	Ns	Ns
Overweight/obesity	2.99 (1.69–5.32)	< 0.0001	2.39 (1.33–4.3)	0.04

_a_Variables excluded from the final model were: physical activity, number of driving hours, waist circumference and professional driving years. _b_Variables excluded from the final model were: age, physical activity, number of driving hours, waist circumference and professional driving years.

## Discussion

The major finding of this study was that male long–distance bus drivers had a higher prevalence of clustering of cardiometabolic risk factors than the general population, and in addition, most them were unaware of their risk status.^12^,^14^ This clustering places them at a higher risk for CVD and contributes significantly to the already burgeoning CVD burden in the general population. Importantly, a CVD event in a driver while driving portends grave danger to him, the passengers and other road users.

The prevalence of hypertension in this study was 39.7%, with 75.9% being newly diagnosed. This is higher than the recent pooled national prevalence rate of 28.9% but lower than the 44.9% prevalence from a national study on blindness and hypertension.^32^,^33^ Previous local studies reported prevalence rates ranging from 21.4 to 33.5%.^19^–^21^ Studies from Brazil and Iran reported prevalence rates of 45.6 and 44.6%, respectively, much higher than their national prevalence rates.^12^,^13^

Professional drivers, by nature of their occupation, are largely sedentary and indulge in dietary indiscretions, which could lead to obesity. From this study, obesity was a predictor of hypertension. Furthermore, BMI and longer duration of years of professional driving significantly correlated with the risk of hypertension, similar to findings by Sangaletti et al.^12^ This association is plausible, as drivers who drive for long hours over many years tend to gain weight inappropriately due to physical inactivity and dietary indiscretion.

In addition to high prevalence of hypertension, optimal blood pressure control was equally low among the subjects. Among the 9.6% previously known hypertensives, only 21.4% had optimal BP control. BP control is generally very low in Nigeria, ranging between five and 29.4%.^34^,^35^ Ignorance, long travel times, poor access to standard medical care, the asymptomatic nature of hypertension and the relative lack of self–care among males have been suggested as possible causes of poor BP control among long–distance drivers.^12^

The prevalence of abnormal glucose profiles in this study was 45.2%, comprising 31.3 and 13.9% for impaired fasting gliucose levels and diabetes mellitus (DM), respectively. Most of the diabetics were diagnosed for the first time during this study. There are no local studies for comparison but the reported prevalence of DM from this study is much higher than the 4.5% reported by the International Diabetes Federation (IDF) and the eight to 10% from a study on the general population.^36^,^37^ In Iran, the prevalence of DM among drivers was 17.5%, comparable to the value obtained from this study, but higher than the 8.5% prevalence reported by the IDF in 2014.^13^ Obesity is a risk factor for type 2 DM. From our study, BMI was a predictor of abnormal glucose profiles, similar to the findings by Sangaletti et al.^12^

The prevalence of dyslipidaemia in this study was 56.3%, comparable to the national average of 60.1%.38 The predominant dyslipidaemia was elevated TC levels in 27.8% of the subjects, followed by elevated LDL–C levels in 24.6%, elevated triglycerides in 24.6% and low HDL–C levels in 6.5%. There are no local studies of lipid abnormalities in professional drivers. The pattern obtained is at variance however with patterns reported in local studies in apparently healthy Nigerians, in which the predominant dyslipidaemia was low HDL–C levels.^38^ In Iran, professional drivers have been shown to have predominantly hypertriglyceridaemia and central obesity, attributable to stressful working conditions.^13^

The combined prevalence of overweight and obesity, measured by BMI in this study, was 62.8%, comparable to the 63.4 and 64.4% reported by similar local studies,^19^–^21^ but higher than the reported prevalence of 31 to 48% in the general Nigerian population.^39^,^40^ Similar international studies documented a prevalence of combined overweight and obesity to be between 62.1 and 78.2%.^13^,^41^,^42^ Using waist circumference, the prevalence of obesity from this study was 24.1%. This was lower than the 58.2 and 63.3% from studies in Brazil and Iran, respectively.^12^,^41^ This difference might be methodological. In these countries the cut–off for abdominal obesity is 88 cm, less than the 102 cm used in our study.^43^,^44^ Prolonged work stress and long hours at work contribute to the development of obesity and abdominal obesity in professional drivers.^13^

The prevalence of physical inactivity in this study was 50.9%, comparable to the 53.4% from a local study,^20^ but lower than the 72.8% reported by similar international studies.^12^,^45^ Both studies were among truck drivers who probably do not have to stop on the way for passengers to alight for refreshments. Physical inactivity and dietary habits of professional drivers are known to predispose to obesity. Obesity increases the risk of hypertension and abnormal glucose profiles, as shown in this study. It is also known to increase the risk of road traffic accidents among professional drivers due to its association with obstructive sleep apnoea and excessive daytime sleepiness, consequent fatigue and reduction in alertness while driving.^46^

The prevalence of smoking in this study was 19.5%. Reported prevalence in similar local studies is between 17.8 and 31.3%, all higher than the 15% in the general population.^20^,^21^,^47^,^48^ The lower prevalence from this study might be due to dilution effect from the ‘no smoking within the bus terminal’ policy of one of the transport companies used in this study. Secondly, the subjects may not have been truthful in their responses to the question on smoking status. Comparable rates of 20 and 15.6% were reported in similar international studies.^12^,^45^

Alcohol consumption was very common in this study group, with a prevalence rate of 71.1%. Reported local prevalence in this group ranged from 34 to 84.4%.^20^,^48^,^49^ These figures are much higher than the 7.6 and 9.1% reported in the general male population.^50^,^51^ A recent local study from Muslim–dominated north–west Nigeria documented a prevalence of 5.5% among inter–city bus drivers.^21^ This very low figure might be related to a religious obligation that forbids Muslims from consuming alcohol.

It is pertinent to note that in this study, CVD risk factors co–occurred, as has been documented in the past.^52^ This clustering of risk factors increases the overall CVD risk of the individual and also makes control difficult due to problems of pill burden.^53^,^54^ In this study 45.1% of the subjects had more than two risk factors clustered together. Clustering of CVD risk factors has been documented in the general population, with prevalence rates between 12.9 and 27.5%, depending on the study population. The commonest risk–factor combinations are hypertension, obesity, abnormal glucose profile and atherogenic dyslipidaemia.^55^–^57^ Our findings are similar to the above pattern, although the combination of hypertension and abnormal glucose level was most prevalent. These findings are similar to the pattern reported in similar studies.^12^,^13^

There were some limitations in this study. The use of glycosylated haemoglobin would have been helpful in assessing the quality of glycaemic control among the diabetic subjects. Bus drivers with poor control of both BP and glucose levels were not assessed for medication adherence.

## Conclusion

Long–distance professional drivers in Nigeria are at a higher risk for CVD than the general male population on account of the higher prevalence of a plethora of risk factors they harbour: hypertension, abnormal glucose profiles, overweight/obesity, alcohol use, smoking and atherogenic dyslipidaemia. These risk factors not only co–occur in a large number of drivers, but most are unaware of their risk. Overweight/obesity is the common driver of hypertension and abnormal glucose profiles among them, while age ≥ 45 years increases the risk of developing hypertension. Contributing to their risk is the social gradient of inequality, which affects their access to healthcare and adherence to medical intervention.

There is therefore a need to increase CVD risk awareness in this vulnerable yet important segment of our population through public awareness campaigns, banning of smoking and selling of alcoholic beverages in motor parks, compulsory annual health screening, defined maximum driving hours per week, provision of facilities to promote physical activity in the motor parks and medical facilities to diagnose, treat and monitor risk–factor control. Universal health insurance coverage as a national health policy would also help in providing healthcare/health promotional services to this group, who at the moment are not covered by the health insurance scheme.
